# (*E*,*E*)-*N*′-{4-[(2-Benzoyl­hydrazin-1-yl­idene)meth­yl]benzyl­idene}benzo­hydrazide

**DOI:** 10.1107/S1600536812014687

**Published:** 2012-04-18

**Authors:** Ramin Karimian, Hassan Hosseini-Monfared, Rahman Bikas, N. Burcu Arslan, Canan Kazak, Ahmet Koroglu

**Affiliations:** a Applied Biotechnology Research Center, Baqiyatallah University of Medical Sciences, Tehran, Iran; b Department of Chemistry, University of Zanjan, 45195-313 Zanjan, Iran; c Young Researchers Club, Tabriz Branch, Islamic Azad University, Tabriz, Iran; d Department of Physics, Faculty of Arts and Sciences, Ondokuz Mayis University, 55019 Kurupelit, Samsun, Turkey

## Abstract

In the title compound, C_22_H_18_N_4_O_2_, the mol­ecules lie across an inversion centre. The dihedral angle between the mean planes of the central and terminal benzene rings is 66.03 (2)°. The mol­ecule displays *trans* and *anti* conformations about the C=N and N—N bonds, respectively. In the crystal, N—H⋯O hydrogen bonds, with the O atoms of C=O groups acting as acceptors, link the mol­ecules into a chain along [101].

## Related literature

For historical background to aroylhydrazones, see: Savanini *et al.* (2002 ▶). For related structures, see: Bikas *et al.* (2012 ▶, 2010*a* ▶,*b* ▶); Hosseini Monfared *et al.* (2010*a* ▶). For catalytic applications of aroylhydrazones, see: Hosseini Monfared *et al.* (2010*b* ▶).
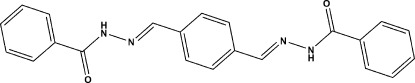


## Experimental

### 

#### Crystal data

C_22_H_18_N_4_O_2_
*M* *_r_* = 370.40
Monoclinic, 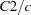

*a* = 30.569 (3) Å
*b* = 5.1845 (3) Å
*c* = 12.5191 (11) Å
β = 112.408 (7)°
*V* = 1834.3 (3) Å^3^
*Z* = 4
Mo *K*α radiation
μ = 0.09 mm^−1^
*T* = 293 K
0.42 × 0.22 × 0.08 mm

#### Data collection

Stoe IPDS 2 diffractometer
Absorption correction: integration (*X-RED32*; Stoe & Cie, 2002 ▶) *T*_min_ = 0.976, *T*_max_ = 0.992
13265 measured reflections
1905 independent reflections
965 reflections with *I* > 2σ(*I*)
*R*_int_ = 0.105

#### Refinement

*R*[*F* ^2^ > 2σ(*F* ^2^)] = 0.051
*wR*(*F* ^2^) = 0.078
*S* = 0.94
1905 reflections
163 parameters
All H-atom parameters refined
Δρ_max_ = 0.11 e Å^−3^
Δρ_min_ = −0.14 e Å^−3^

### 

Data collection: *X-AREA* (Stoe & Cie, 2002 ▶); cell refinement: *X-AREA*; data reduction: *X-RED32* (Stoe & Cie, 2002 ▶); program(s) used to solve structure: *SHELXS97* (Sheldrick, 2008) ▶; program(s) used to refine structure: *SHELXL97* (Sheldrick, 2008) ▶; molecular graphics: *ORTEP-3 for Windows* (Farrugia, 1997 ▶); software used to prepare material for publication: *WinGX* (Farrugia, 1999 ▶) and *PLATON* (Spek, 2009 ▶).

## Supplementary Material

Crystal structure: contains datablock(s) I, global. DOI: 10.1107/S1600536812014687/qm2059sup1.cif

Structure factors: contains datablock(s) I. DOI: 10.1107/S1600536812014687/qm2059Isup2.hkl

Supplementary material file. DOI: 10.1107/S1600536812014687/qm2059Isup3.cml

Additional supplementary materials:  crystallographic information; 3D view; checkCIF report

## Figures and Tables

**Table 1:** Hydrogen-bond geometry (Å, °)

*D*—H⋯*A*	*D*—H	H⋯*A*	*D*⋯*A*	*D*—H⋯*A*
N1—H1⋯O1^i^	0.87 (2)	2.19 (2)	3.056 (3)	171 (2)

Symmetry code: (i) 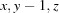.

**Fig. 1.**
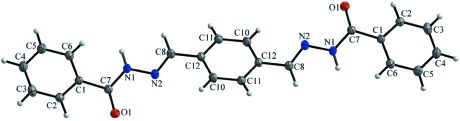
The molecular structure of the title compound. Displacement ellipsoids are drawn at the 30% probability level.

**Fig. 2.**
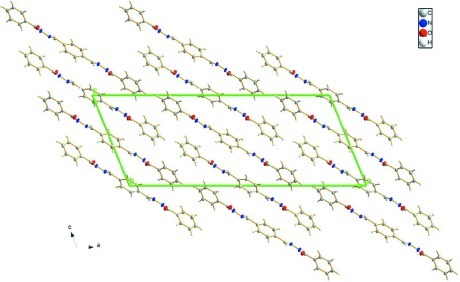
The packing diagram of the title compound.

**Fig. 3.**
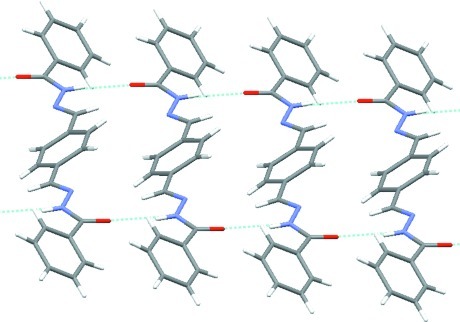
Hydrogen bonding in the title compound. The blue dashed lines indicate intermolecular N–H···O hydrogen bonds.

## supplementary crystallographic information

### Comment

The design, synthesis, and characterization of metal complexes with Schiff-base ligands play a vital role in the coordination chemistry of transition metals. Hydrazones are a special group of compounds in the Schiff base family that are characterized by the presence of *RR*'C=N–N=C(O)*R*'' which two inter-linked nitrogen atoms (–N—N–) separate them from imines, oximes, *etc*. Hydrazone ligands derived from the condensation of acid hydrazides (R–CO–NH–NH_2_) with aromatic carbonyl compounds are important O, N-donor ligands. As biologically active compounds, hydrazones find application in the treatment of diseases such as tuberculosis, leprosy and mental disorder and also as anti-tumor agents. Hydrazone Schiff bases also have wide spread applications in fields such as coordination chemistry, bioinorganic chemistry , magnetics, electronics, nonlinear optics and fluorescent materials. Aroylhydrazone complexes also seem to be good candidates for catalytic oxidation studies because of their resistance to oxidation (Hosseini Monfared *et al.*, 2010*b*). As part of our studies on the synthesis and characterization of hydrazone derivatives (Bikas *et al.*, 2012, 2010**a*,b*), we report here the crystal structure of (*N',N''E,N',N''E*)-*N',N''*-(1,4-phenylenebis(methan-1-yl-1-ylidene))dibenzohydrazide. The molecules of C_22_H_18_N_4_O_2_, lie across inversion centres and the asymmetric unit contains a half molecule (Fig. 1). The terminal benzene rings are parallel to each other and the distance of two planes which embrace these rings is 3.168 Å apart. The dihedral angle between the mean planes of the central and two terminal benzene rings is 66.03 (2)°. The molecule displays a *trans* configuration with respect to the C=N and N—N bonds. The packing diagram of the title compound is shown in Fig. 2. There are two strong intermolecular N—H···O hydrogen bonds in which the O atoms of the carbonyl groups (–C=O) act as hydrogen acceptors for the hydrogen of N—H and a one-dimensional chain is formed by these hydrogen bonds (Fig. 3).

### Experimental

For preparing the title compound a methanol (10 ml) solution of benzhydrazide (3 mmol) was added drop-wise to a methanol solution (10 ml) of terephthalaldehyde (1.5 mmol), and the mixture was refluxed for 4 h. The solution was then evaporated on a steam bath to 5 *cm*^3^ and cooled to room temperature. The white precipitates of the title compound were separated and filtered off, washed with 3 ml of cooled methanol and then dried in air. Colorless crystals were obtained from its methanol solution by thermal gradient method. Yield: 93%. IR (cm^-1^): 3253 (s, broad, N—H), 1654 (*vs*, C=O), 1607 (*s*, C=N), 1546 (*vs*), 1507 (*m*), 1361 (*s*), 1284 (*vs*), 1146 (*s*), 1069 (*s*), 969 (*m*), 915 (*m*), 846 (*m*), 723 (*s*), 692 (*s*), 661 (*s*), 569 (*w*), 538 (*w*), 423 (*w*).

### Crystal data

**Table:**

C_22_H_18_N_4_O_2_	*F*(000) = 776
*M**_r_* = 370.40	*D*_x_ = 1.341 Mg m^−^^3^
Monoclinic, *C*2/*c*	Mo *K*α radiation, λ = 0.71073 Å
Hall symbol: -C 2yc	Cell parameters from 8004 reflections
*a* = 30.569 (3) Å	θ = 1.4–27.5°
*b* = 5.1845 (3) Å	µ = 0.09 mm^−^^1^
*c* = 12.5191 (11) Å	*T* = 293 K
β = 112.408 (7)°	Prism, colorless
*V* = 1834.3 (3) Å^3^	0.42 × 0.22 × 0.08 mm
*Z* = 4	

### Data collection

**Table:**

Stoe IPDS 2 diffractometer	1905 independent reflections
Radiation source: fine-focus sealed tube	965 reflections with *I* > 2σ(*I*)
Plane graphite monochromator	*R*_int_ = 0.105
Detector resolution: 6.67 pixels mm^-1^	θ_max_ = 26.5°, θ_min_ = 1.4°
rotation method scans	*h* = −38→38
Absorption correction: integration (*X-RED32*; Stoe & Cie, 2002)	*k* = −6→6
*T*_min_ = 0.976, *T*_max_ = 0.992	*l* = −15→15
13265 measured reflections	

### Refinement

**Table:**

Refinement on *F*^2^	Primary atom site location: structure-invariant direct methods
Least-squares matrix: full	Secondary atom site location: difference Fourier map
*R*[*F*^2^ > 2σ(*F*^2^)] = 0.051	Hydrogen site location: inferred from neighbouring sites
*wR*(*F*^2^) = 0.078	All H-atom parameters refined
*S* = 0.94	*w* = 1/[σ^2^(*F*_o_^2^) + (0.0207*P*)^2^] where *P* = (*F*_o_^2^ + 2*F*_c_^2^)/3
1905 reflections	(Δ/σ)_max_ = 0.001
163 parameters	Δρ_max_ = 0.11 e Å^−^^3^
0 restraints	Δρ_min_ = −0.14 e Å^−^^3^

### Special details

**Table:**

Geometry. All e.s.d.'s (except the e.s.d. in the dihedral angle between two l.s. planes) are estimated using the full covariance matrix. The cell e.s.d.'s are taken into account individually in the estimation of e.s.d.'s in distances, angles and torsion angles; correlations between e.s.d.'s in cell parameters are only used when they are defined by crystal symmetry. An approximate (isotropic) treatment of cell e.s.d.'s is used for estimating e.s.d.'s involving l.s. planes.
Refinement. Refinement of *F*^2^ against ALL reflections. The weighted *R*-factor *wR* and goodness of fit *S* are based on *F*^2^, conventional *R*-factors *R* are based on *F*, with *F* set to zero for negative *F*^2^. The threshold expression of *F*^2^ > σ(*F*^2^) is used only for calculating *R*-factors(gt) *etc*. and is not relevant to the choice of reflections for refinement. *R*-factors based on *F*^2^ are statistically about twice as large as those based on *F*, and *R*- factors based on ALL data will be even larger.

### Fractional atomic coordinates and isotropic or equivalent isotropic displacement parameters (Å^2^)

**Table:**

	*x*	*y*	*z*	*U*_iso_*/*U*_eq_	
C1	0.84753 (7)	0.1985 (4)	0.32678 (19)	0.0454 (6)	
C2	0.84744 (9)	0.3457 (5)	0.4191 (2)	0.0562 (7)	
C3	0.81956 (9)	0.2794 (6)	0.4790 (2)	0.0658 (8)	
C4	0.79059 (10)	0.0693 (6)	0.4454 (3)	0.0695 (8)	
C5	0.78956 (10)	−0.0782 (5)	0.3532 (3)	0.0711 (8)	
C6	0.81817 (8)	−0.0140 (5)	0.2948 (2)	0.0582 (7)	
C7	0.87799 (7)	0.2804 (4)	0.26408 (19)	0.0475 (6)	
C8	0.94196 (8)	−0.0556 (5)	0.1321 (2)	0.0502 (6)	
C10	0.96656 (9)	0.1846 (5)	−0.0081 (2)	0.0558 (7)	
C11	1.00525 (9)	−0.2099 (5)	0.0707 (2)	0.0553 (7)	
C12	0.97114 (7)	−0.0250 (4)	0.06315 (18)	0.0458 (6)	
N1	0.89465 (7)	0.0866 (4)	0.21865 (17)	0.0543 (6)	
N2	0.92141 (7)	0.1407 (3)	0.15428 (16)	0.0516 (5)	
O1	0.88701 (6)	0.5082 (3)	0.25515 (14)	0.0650 (5)	
H1	0.8912 (7)	−0.074 (4)	0.2351 (18)	0.055 (7)*	
H2	0.9404 (6)	−0.232 (4)	0.1624 (16)	0.052 (6)*	
H3	0.8174 (7)	−0.118 (4)	0.2312 (19)	0.058 (7)*	
H4	0.9424 (8)	0.305 (4)	−0.0167 (18)	0.065 (7)*	
H5	1.0094 (7)	−0.343 (4)	0.1209 (18)	0.056 (7)*	
H6	0.8219 (8)	0.387 (4)	0.546 (2)	0.078 (8)*	
H7	0.8670 (7)	0.499 (4)	0.4386 (17)	0.068 (7)*	
H8	0.7688 (9)	−0.228 (5)	0.324 (2)	0.101 (10)*	
H9	0.7717 (9)	0.017 (5)	0.489 (2)	0.096 (9)*	

### Atomic displacement parameters (Å^2^)

**Table:**

	*U*^11^	*U*^22^	*U*^33^	*U*^12^	*U*^13^	*U*^23^
C1	0.0450 (14)	0.0457 (13)	0.0519 (16)	0.0066 (11)	0.0255 (13)	0.0039 (12)
C2	0.0568 (16)	0.0572 (16)	0.0627 (18)	−0.0026 (13)	0.0319 (15)	−0.0062 (13)
C3	0.0676 (17)	0.0798 (19)	0.0621 (19)	0.0055 (16)	0.0385 (17)	−0.0027 (16)
C4	0.0691 (19)	0.0738 (19)	0.084 (2)	0.0066 (15)	0.0495 (18)	0.0112 (17)
C5	0.0715 (19)	0.0601 (17)	0.099 (2)	−0.0068 (15)	0.0518 (19)	−0.0044 (17)
C6	0.0618 (15)	0.0521 (14)	0.0735 (19)	−0.0023 (13)	0.0401 (15)	−0.0111 (15)
C7	0.0477 (13)	0.0479 (14)	0.0513 (16)	0.0053 (12)	0.0239 (12)	0.0012 (12)
C8	0.0532 (15)	0.0486 (16)	0.0569 (16)	0.0007 (12)	0.0301 (13)	−0.0008 (12)
C10	0.0580 (16)	0.0553 (15)	0.0662 (18)	0.0137 (13)	0.0375 (15)	0.0052 (13)
C11	0.0655 (16)	0.0507 (14)	0.0605 (18)	0.0083 (13)	0.0362 (15)	0.0045 (13)
C12	0.0454 (14)	0.0483 (13)	0.0504 (15)	−0.0004 (12)	0.0259 (12)	−0.0059 (12)
N1	0.0648 (14)	0.0476 (12)	0.0704 (15)	0.0036 (11)	0.0481 (13)	0.0054 (11)
N2	0.0531 (12)	0.0537 (12)	0.0599 (13)	0.0006 (9)	0.0349 (11)	−0.0002 (10)
O1	0.0857 (12)	0.0445 (9)	0.0850 (13)	−0.0003 (9)	0.0552 (10)	0.0020 (9)

### Geometric parameters (Å, º)

**Table:**

C1—C6	1.381 (3)	C7—N1	1.346 (3)
C1—C2	1.386 (3)	C8—N2	1.281 (3)
C1—C7	1.490 (3)	C8—C12	1.467 (3)
C2—C3	1.377 (3)	C8—H2	1.00 (2)
C2—H7	0.97 (2)	C10—C11^i^	1.375 (3)
C3—C4	1.365 (4)	C10—C12	1.379 (3)
C3—H6	0.99 (2)	C10—H4	0.94 (2)
C4—C5	1.374 (3)	C11—C10^i^	1.375 (3)
C4—H9	0.98 (3)	C11—C12	1.392 (3)
C5—C6	1.378 (3)	C11—H5	0.91 (2)
C5—H8	0.98 (3)	N1—N2	1.378 (2)
C6—H3	0.95 (2)	N1—H1	0.87 (2)
C7—O1	1.228 (2)		
			
C6—C1—C2	118.3 (2)	O1—C7—C1	121.95 (19)
C6—C1—C7	122.8 (2)	N1—C7—C1	115.0 (2)
C2—C1—C7	118.9 (2)	N2—C8—C12	120.0 (2)
C3—C2—C1	121.0 (3)	N2—C8—H2	123.2 (11)
C3—C2—H7	121.3 (13)	C12—C8—H2	116.7 (11)
C1—C2—H7	117.7 (13)	C11^i^—C10—C12	120.8 (2)
C4—C3—C2	119.6 (3)	C11^i^—C10—H4	120.5 (13)
C4—C3—H6	122.6 (14)	C12—C10—H4	118.5 (13)
C2—C3—H6	117.8 (14)	C10^i^—C11—C12	120.8 (2)
C3—C4—C5	120.5 (3)	C10^i^—C11—H5	120.9 (13)
C3—C4—H9	120.0 (15)	C12—C11—H5	118.2 (13)
C5—C4—H9	119.4 (15)	C10—C12—C11	118.4 (2)
C4—C5—C6	119.7 (3)	C10—C12—C8	122.1 (2)
C4—C5—H8	124.0 (17)	C11—C12—C8	119.5 (2)
C6—C5—H8	116.3 (17)	C7—N1—N2	120.0 (2)
C5—C6—C1	120.8 (3)	C7—N1—H1	120.9 (14)
C5—C6—H3	119.3 (13)	N2—N1—H1	118.9 (14)
C1—C6—H3	119.9 (13)	C8—N2—N1	114.51 (19)
O1—C7—N1	123.0 (2)		
			
C6—C1—C2—C3	1.3 (4)	C2—C1—C7—N1	149.0 (2)
C7—C1—C2—C3	179.4 (2)	C11^i^—C10—C12—C11	0.4 (4)
C1—C2—C3—C4	−1.7 (4)	C11^i^—C10—C12—C8	179.2 (2)
C2—C3—C4—C5	0.9 (4)	C10^i^—C11—C12—C10	−0.4 (4)
C3—C4—C5—C6	0.3 (4)	C10^i^—C11—C12—C8	−179.3 (2)
C4—C5—C6—C1	−0.7 (4)	N2—C8—C12—C10	−20.1 (3)
C2—C1—C6—C5	−0.1 (4)	N2—C8—C12—C11	158.7 (2)
C7—C1—C6—C5	−178.1 (2)	O1—C7—N1—N2	−3.0 (3)
C6—C1—C7—O1	147.0 (2)	C1—C7—N1—N2	177.04 (19)
C2—C1—C7—O1	−30.9 (3)	C12—C8—N2—N1	179.3 (2)
C6—C1—C7—N1	−33.0 (3)	C7—N1—N2—C8	168.7 (2)

Symmetry code: (i) −*x*+2, −*y*, −*z*.

### Hydrogen-bond geometry (Å, º)

**Table:**

*D*—H···*A*	*D*—H	H···*A*	*D*···*A*	*D*—H···*A*
N1—H1···O1^ii^	0.87 (2)	2.19 (2)	3.056 (3)	171 (2)

Symmetry code: (ii) *x*, *y*−1, *z*.
